# Microbial community composition and diversity in the Indian Ocean deep sea REY-rich muds

**DOI:** 10.1371/journal.pone.0208230

**Published:** 2018-12-17

**Authors:** Shuyan Wang, Miao Yu, Jiaqiang Wei, Mu Huang, Xuefa Shi, Hao Chen

**Affiliations:** 1 Key Lab of Marine Bioactive Substances of SOA, The First Institute of Oceanography, Qingdao, China; 2 Laboratory for Marine Biology and Biotechnology, Qingdao National Laboratory for Marine Science and Technology, Qingdao, China; 3 Qingdao Key Lab of Marine Natural Products R&D, Qingdao, China; 4 Laboratory for Marine Geology, Qingdao National Laboratory for Marine Science and Technology, Qingdao, China; National Cheng Kung University, TAIWAN

## Abstract

Studies about the composition and diversity of microbial community in the Rare Earth Elements-rich muds are limited. In this research, we conducted a characterization for the composition and diversity of bacterial and archaeal communities from rare earth elements-rich gravity core sediment at approximately 4800 meters deep in the Indian Ocean by Illumina high-throughput sequencing targeting 16S rRNA genes. The results showed that the most abundant bacteria were Proteobacteria, followed by Firmicutes and Actinobacteria. Amongst Proteobacteria, Gammaproteobacteria are present in all sections of this sediment core accounted for a particularly large proportion of bacterial sequences. *Candidatus Nitrosopumilus*, with a higher relative abundance in our samples, belongs to Thaumarchaeota. This is the first report on the composition and diversity of rare earth elements-rich muds microbial communities in the Indian Ocean deep sea.

## 1 Introduction

Rare earth elements (REY) are comprised of the lanthanides (atomic numbers 57–71), scandium (atomic number 21) and yttrium (atomic number 39) in the periodic table. Due to its special atomic structure and active chemical properties, REY, by combining other elements, can produce various advanced materials and then widely applied to agricultural activities [1] and global usage of technological devices such as superconductors, magnets, rechargeable batteries, cell phones, fluorescent monitors and so on [2]. With the increasing demand for REY and the growing importance of REY as a new type of strategic mineral resource, REY have attracted extensive attentions worldwide in recent years.

Rare earths had remained undisturbed in the deep-sea environment from an earlier, yet it was not until 2011 that Yasuhiro Kato reported deep-sea mud contains high concentrations of these elements in the eastern South and central North Pacific [3]. Additionally, given the limited research on the rare earth minerals distributed in seafloor, the formation mechanism of these deposits in deep-water is still an unknown scientific issue up to now.

Microbes are ubiquitous in almost all environments and play pivotal roles in the biogeochemical process. Previously published studies have primarily investigated how microbial communities are formed under markedly different terrestrial or aquatic ecosystems [4–9], yet such researches in abyssal regions are scarce even though pelagic sediments harbor relatively large numbers of microorganisms, the integral constituents of the biogeochemical cycles. Especially in the REY-rich muds of submarine areas, while there have been studies into the geochemistry and mineralogy aspects of the special regions [10, 11], the microbial ecology of the abyssal REY-rich muds has never been characterized. Simultaneously, the metabolic activities of microbial populations have vital effects on the metallogenic processes of submarine mineral resources, but, insight into the functions of microorganisms on the formation of deep-sea rare earth minerals is still significantly lagging behind that into other types of submarine mineral resources (such as polymetallic nodules [12], cobalt-rich crusts [13], and polymetallic sulfides). As a result, there has been little knowledge on the interaction between the structure of microbial communities and the formations of seafloor rare earths minerals.

In order to fill the research gap described above, we conducted a characterization for the composition and diversity of bacterial and archaeal communities in REY-rich muds in the Indian Ocean via Illumina high-throughput sequencing targeting 16S rRNA genes. The primary aim of this study is to investigate the taxonomic profile of microbial communities and determine the influence of various environmental factors on the diversity of microbial communities. The secondary aim is to provide reliable data for further research on relationship between microorganisms and rare earth minerals in the deep-sea. This current study is one of the first research into the microbial communities in REY-rich muds in the central Indian Ocean basin and propose a biological perspective to complementally explain the process of REY mineralization in the seafloor.

## 2 Materials and methods

### 2.1 Sample collection and edaphic properties measurement

Samples were collected from the 34Ⅴ cruise that was organized by the China Ocean Mineral Resources R & D Association in the summer of 2015. A 270 cm-length of sediment gravity core was obtained below 4888 m sea level at an abyssal basin area in the central Indian Ocean (22.47°S, 81.40°E), which contains remarkably high concentrations of REY that has not been found in the past (Table 1). After the collection, intact core was sealed and stored at 4°C for about three weeks, then was sectioned from the top to the bottom at 10 cm depth intervals. After numbering them from GC05.1 to GC05.27, we acquired 27 samples representing individually perpendicular depths sediments for this study, ultimately.

**Table 1:** The REY content of all samples.

sample name	La	Ce	Pr	Nd	Sm	Eu	Gd	Tb	Dy	Ho	Er	Tm	Yb	Lu	Y	Sc	TREY^a^
	(10^−6^)	(10^−6^)	(10^−6^)	(10^−6^)	(10^−6^)	(10^−6^)	(10^−6^)	(10^−6^)	(10^−6^)	(10^−6^)	(10^−6^)	(10^−6^)	(10^−6^)	(10^−6^)	(10^−6^)	(10^−6^)	(10^−6^)
**GC05.1**	100	200	27.0	120	27.4	6.67	26.5	4.39	28.9	5.15	14.4	2.24	13.6	2.16	172	22.5	773.42
**GC05.2**	106	201	28.0	123	28.5	6.94	27.2	4.49	30.1	5.28	14.8	2.33	14.2	2.26	183	22.6	799.59
**GC05.3**	119	216	31.8	142	32.6	7.97	31.5	5.22	34.4	6.12	16.9	2.70	16.1	2.60	210	25.2	899.73
**GC05.4**	118	224	34.5	147	34.4	8.93	32.9	5.99	37.1	6.10	16.9	2.86	17.5	2.54	200	24.4	912.65
**GC05.5**	125	226	33.4	147	33.9	8.35	32.5	5.44	36.0	6.37	17.7	2.76	16.9	2.66	220	27.0	941.27
**GC05.6**	124	222	33.4	148	34.0	8.35	32.8	5.43	35.8	6.35	17.5	2.71	16.7	2.66	216	25.9	932.17
**GC05.7**	96	195	28.4	120	27.8	7.19	26.6	4.86	30.1	5.69	15.6	2.36	14.2	2.35	160	20.3	756.6
**GC05.8**	140	224	34.1	150	34.4	8.46	33.3	5.50	36.4	6.43	18.0	2.79	16.9	2.72	220	26.1	959.06
**GC05.9**	127	229	36.2	152	34.6	8.35	33.3	5.70	34.4	6.16	16.5	2.64	16.0	2.57	192	25.9	922.59
**GC05.10**	149	225	36.2	161	37.1	9.00	35.2	5.86	39.1	6.94	19.2	3.04	18.4	2.93	237	24.9	1009.92
**GC05.11**	136	226	36.2	161	36.8	8.97	35.3	5.94	39.3	6.99	19.6	3.06	18.6	2.99	240	25.4	1001.46
**GC05.12**	125	202	34.9	146	33.4	8.40	32.3	5.82	35.2	6.59	18.4	2.82	17.1	2.70	222	26.0	917.84
**GC05.13**	138	219	39.7	169	39.0	10.19	37.9	6.86	43.3	8.21	22.4	3.37	20.4	3.36	219	23.7	1003.45
**GC05.14**	172	225	41.3	185	41.9	10.30	40.7	6.84	45.4	8.14	22.6	3.53	21.2	3.40	284	28.7	1138.92
**GC05.15**	192	228	46.1	207	46.8	11.33	45.4	7.63	50.0	9.11	25.4	3.91	23.7	3.81	317	29.3	1246.77
**GC05.16**	182	231	52.6	227	51.4	13.44	49.7	9.12	57.8	11.14	30.2	4.55	27.2	4.40	297	26.8	1275.82
**GC05.17**	262	230	56.0	280	62.0	15.20	61.3	10.34	70.1	12.54	34.8	5.40	32.4	5.13	446	30.8	1614.28
**GC05.18**	281	227	60.5	293	64.3	15.95	64.2	10.81	73.4	13.34	37.4	5.79	34.8	5.50	487	33.7	1706.83
**GC05.19**	289	234	72.4	295	64.9	15.94	65.2	10.92	75.3	13.81	38.6	6.01	36.4	5.71	514	34.8	1771.75
**GC05.20**	277	230	43.6	280	61.4	15.12	61.5	10.34	71.7	13.11	36.9	5.77	35.2	5.58	494	35.5	1675.8
**GC05.21**	265	227	58.7	269	59.0	14.43	59.5	10.05	68.2	12.60	36.1	5.71	34.1	5.50	475	33.3	1633.19
**GC05.22**	249	220	54.0	252	54.7	13.43	54.7	9.18	61.8	11.57	32.8	5.12	31.2	5.03	445	34.1	1533.6
**GC05.23**	231	217	52.4	237	51.7	12.65	51.6	8.56	58.3	10.77	30.6	4.79	29.0	4.69	410	32.4	1443.12
**GC05.24**	225	217	42.3	229	50.1	12.20	49.6	8.31	56.4	10.32	29.4	4.59	27.7	4.51	397	32.4	1396.01
**GC05.25**	189	229	51.3	218	48.4	12.52	47.6	8.80	56.3	10.92	30.4	4.63	28.0	4.56	311	27.9	1277.99
**GC05.26**	207	225	46.3	208	45.9	11.08	45.0	7.54	51.3	9.35	26.6	4.23	25.4	4.04	346	30.0	1292.26
**GC05.27**	175	224	47.8	206	45.8	11.70	44.3	8.16	51.5	10.06	28.2	4.28	26.0	4.17	282	26.0	1195.29

^a^TREY, the sum of all kinds of rare earth elements.

Subsequently, all collections were divided into two parts. One part was measured for analysis of sediments physicochemical properties according to the methods described by Jingjing GAO [14], including total nitrogen (TN), total carbon (TC), moisture content (MC), REY contents, some major element oxides contents and trace elements contents. The other part aiming to be analyzed microbial data were packed in sterile plastic bags and then frozen at -80°C in the refrigerator prior to nucleic acid extraction.

### 2.2 DNA extraction, PCR amplification and Illumina HiSeq sequencing

Genomic DNA was extracted from each 0.5 g subsample of each section with the exception of the uppermost GC05.1 using the MoBio PowerSoil DNA isolation kit (MoBio Laboratories Inc., Carlsbad, CA, USA) according to the manufacturer’s instructions. After checking DNA concentration and purity, the DNA extracts were used for the subsequent PCR and sequencing experiments. Briefly, bacteria-targeted PCR amplifications were conducted with the specific primer sets 314F/806R (CCTACGGGRBGCASCAG—GGACTACNNGGGTATCTAAT) [15], as were archaea using Arch519F/Arch915R (CAGCCGCCGCGGTAA—GTGCTCCCCCGCCAATTCCT) [16], which respectively amplifies the V3-V4 and V4-V5 hyper-variable regions of the 16S rRNA genes with the reverse primers modified to contain an 8-bp error-correcting barcode unique to each sample. All PCR reactions were performed in triplicate according to the protocols described by Shuo Jiao [4]. Consequently, the triplicate PCR products were pooled together and detected by electrophoresis in agarose gels (2%, w/v).

All amplicons with bright band between 400 bp and 450 bp were chosen and combined in equimolar concentrations for purification using a GeneJET Gel Extraction Kit (Thermo Fisher Scientific, Carlsbad, CA, USA). Furthermore, sequencing libraries were generated using the Illumina TruSeq DNA PCR-FREY Library Preparation Kit (New England Biolabs, Ipswich, MA, USA) following manufacturer’s recommendations, and index codes were added. After assessing the library quality, the library was sequenced on an Illumina HiSeq platform and 250 bp paired-end reads were generated at the Novogene Bioinformatics Technology Co., Ltd. (Tianjin, China).

### 2.3 Data analysis

Paired-end reads were assigned to each sample based on their unique barcode and truncated by cutting off the barcode and primer sequence. After initial trimming, we obtained the raw tags merged by FLASH (V1.2.7) [17]. In order to make the results more accurate and reliable, raw data required qualities filtration with the QIIME (V1.7.0) [18] and Chimeras removal with the UCHIME algorithm [19]. Finally, we generated high-quality clean data that could be used for downstream analyses.

Based on these effective data, sequences were classified into the same operational taxonomic units (OTUs) at an identity threshold of 97% similarity using UPARSE software (V7.0.1001) [20]. Meanwhile, a representative sequence was screened via selecting the longest sequence that had the largest number of hits to other sequences in each OTU. For bacteria, each representative sequence was annotated taxonomic information by the Mothur and assigned to taxonomic groups using the SILVA SSU database (http://www.arb-silva.de/) [21, 22]. For archaea, the representative sequence was determined through RDP classifier (V2.2) [23], followed by aligned using the GreenGene Database (http://greengenes.secondgenome.com/downloads/database/13_5) [24].

To study phylogenetic relationships of different OTUs, and the differences of the dominant species in different samples (groups), multiple sequence alignments were conducted by the MUSCLE software (V 3.8.3) [25] at this point. With regard to subsequent statistical analysis, alpha diversity was applied in analyzing complexity of species diversity for a sample via 6 indices, including Observed-species, Chao1, Shannon, Simpson, ACE, and Good-coverage through the QIIME software (V1.7.0) [18]. And beta diversity analysis, such as principal coordinate analysis (PCoA), was used to evaluate differences of samples in species complexity both on weighted and unweighted Unifrac distance matrices [26]. The relevance of environmental factors in explaining the distribution patterns of microbial communities was analyzed by redundancy analysis (RDA) using R software (V 2.15.3) with the aid of the package’s vegan. To test the correlations between the microbial community diversity and environmental factors, we adopt the Spearman correlation analysis method that was also performed with the R software (V 2.15.3).

## 3 Results

### 3.1 Physicochemical characteristics of sediments

**Fig 1:**
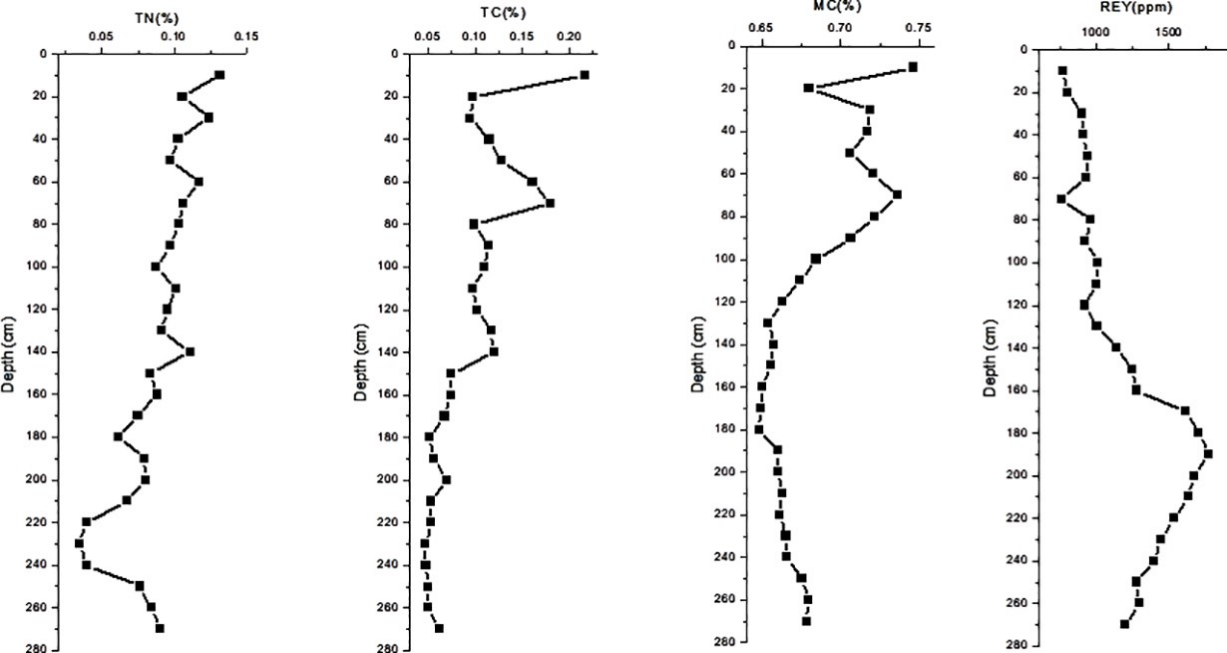
Depth profiles of TN, TC, MC and REY contents at the studied site. TN, total nitrogen; TC, total carbon; MC, moisture content; REY, rare earth elements.

The sediments types were eupelagic clay (0-160cmbsf) to zeolite clay (170-270cmbsf). The data of all kinds of REY contents in each sample were displayed in Table 1. And the major physicochemical properties such as the sediment nutrients contents (TN, TC, TOC), moisture content (MC), major elements oxides contents (SiO_2_, Al_2_O_3_, CaO, Fe_2_O_3_, K_2_O, MgO, MnO, Na_2_O, P_2_O_5_, TiO_2_), partial trace elements contents (Ba, Sr, V, Cu, Ni, Pb, Co, Zn) were summarized in S1 Table. In particular, the data of total nitrogen (TN), total carbon (TC), moisture content (MC) and rare earth elements (REY) were shown in Fig 1.

For sediment nutrients contents profile of this sampling site, along with increasing depths, the content of TN has declined in fluctuation, and the content of TC and MC showed a downward trend on the whole but they could be roughly divided into two sections with 140 or 100 cm below seafloor (cmbsf) as the boundary respectively: the percentages of content in the upper layer was higher and the magnitude of volatility in the lower layer was smaller. On the contrary to the nutrient’s contents, the REY concentration was increased along with increasing depths and was extremely high after the 170 cmbsf of the core sediments.

We also found slight variation of other physicochemical properties in the vertical gradients (S1 Table), as shown by the increase in most major elements oxides contents with the exception of SiO_2_ and Na_2_O, as well as in some trace elements contents at the exclusion of Ba. Although there were minor vertical discrepancies of physicochemical properties in different depths, overall, all the sampled sediments were fairly homogeneous.

With respect to detailed concentrations of REY content in our samples, we can see that all the samples contained 16 kinds of REY (Table 1). But the contents of REY are different in allopelagic sediments samples. Except for Ce, the concentrations of other REY increased at the deeper depths. Especially at the 190 cmbsf of the core sediments, the total of REY content (TREY) reached the highest value that was 1771.75 ppm.

### 3.2 Basic statistics of sequences

Since the top sample might be contaminated during the process of sediment collection, we gave up the sample GC05.1 for bacterial and archaeal downstream analyses. And for bacteria, samples GC05.4, GC05.20 and GC05.24 DNA concentrations were too low to be checked on agarose gels, yielding a total of 23 samples that were used for subsequent experiments. Samples GC05.6 and GC05.26 were also excluded from the archaeal samples due to the same situation.

After sequencing with the Illumina HiSeq and quality control procedures, we obtained 1,117,852 of V3–V4 16S rRNA quality sequences from the 23 bacteria samples finally. The mean number of sequences per sample was 48,602 (min = 21,604, max = 73,534), and the average length of the effective sequences reads was 422 bp. The total OTU number was 18,985 with a sequence similarity of 97%, and then these OTUs were classified into 49 phyla and 585 genera. Equally, after a series of data processing, the high-throughput sequencing of the 24 archaeal sediments resulted in 1,283,303 of V4–V5 16S rRNA effective sequences averaging 53,471 sequences for each sample (min = 34,464, max = 69,372), with the average length of the 382 bp. A total of 4,886 OTUs determined at 97% sequences similarity were identified across the samples examined only into 6 phyla and 4 genera. The bacterial and archaeal sequence data generated in this study were deposited in the NCBI Sequence Read Archive under the accession numbers SRP150581 and SRP150655, respectively.

Rarefaction curves, which show the observed OTUs richness as a function of sequencing effort, have almost approached the saturation plateau for all samples, therefore, it is reasonable to conclude that the sequencing depth was sufficient to capture most microbial diversity information at present (S1 Fig).

### 3.3 Bacterial and archaeal community composition

**Fig 2:**
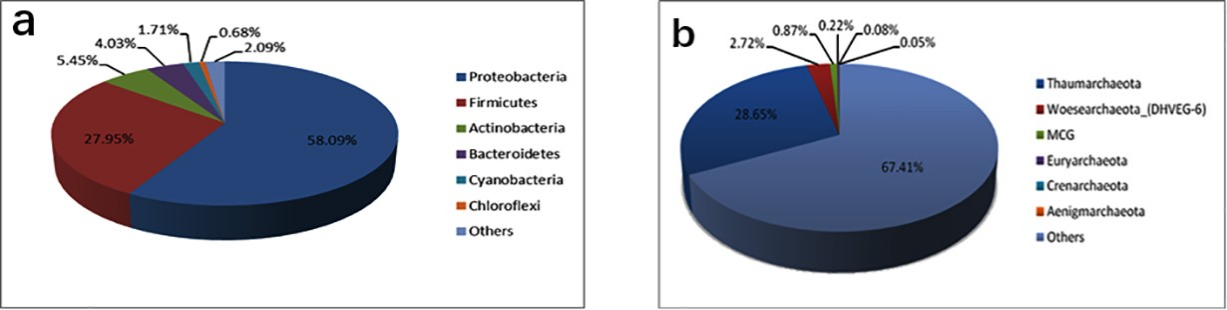
Majority of sequences belonging to different bacteria a)/archaea b) phyla in core sediment.

49 different bacteria phyla or 6 archaea phyla were detected across all sediment samples. For better understanding of the dominant species at the phylum level among these samples, we selected the top six predominant bacteria phyla that contained the majority proportion of bacterial sequences and all detected archaea phyla to make pie charts respectively (Fig 2)

**Fig 3:**
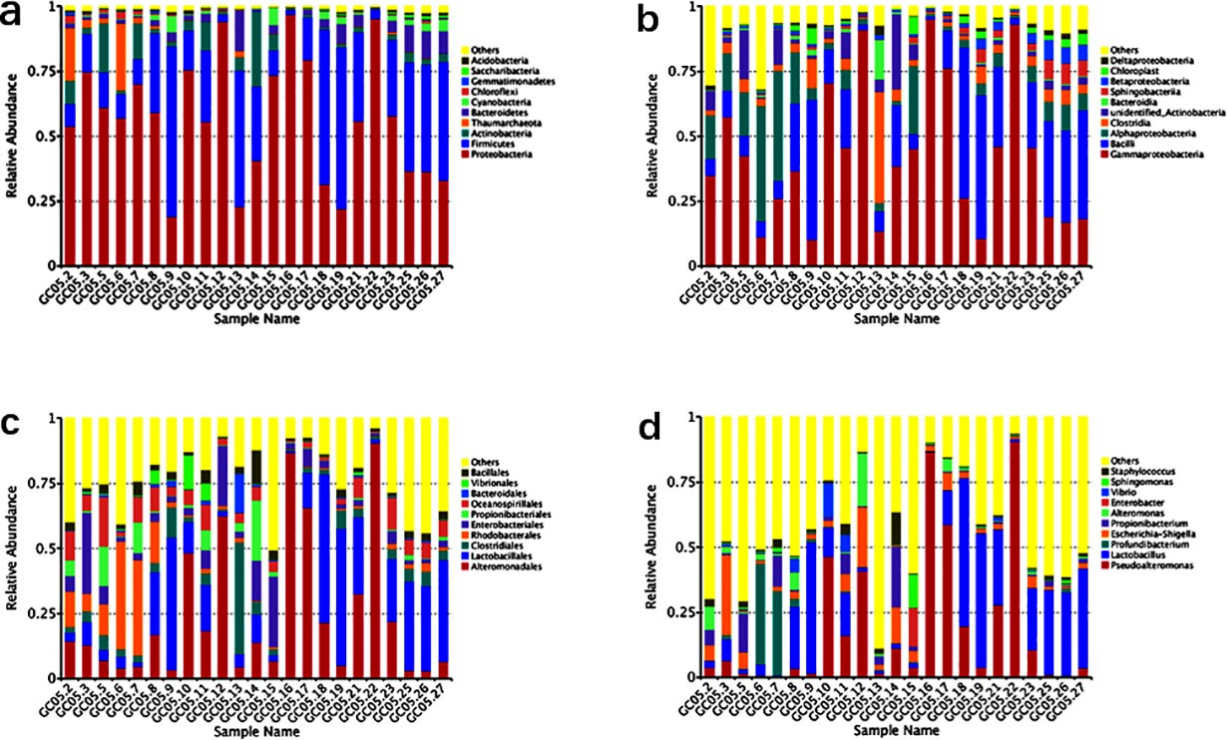
Relative abundance of dominant bacterial groups observed in sediments. Taxonomic distributions are depicted for the ranks of Phylum a), Class b), Order c) and Genera d). Each bar represents the relative abundance of each sample and each color represents a particular bacterial specie.

**Fig 4:**
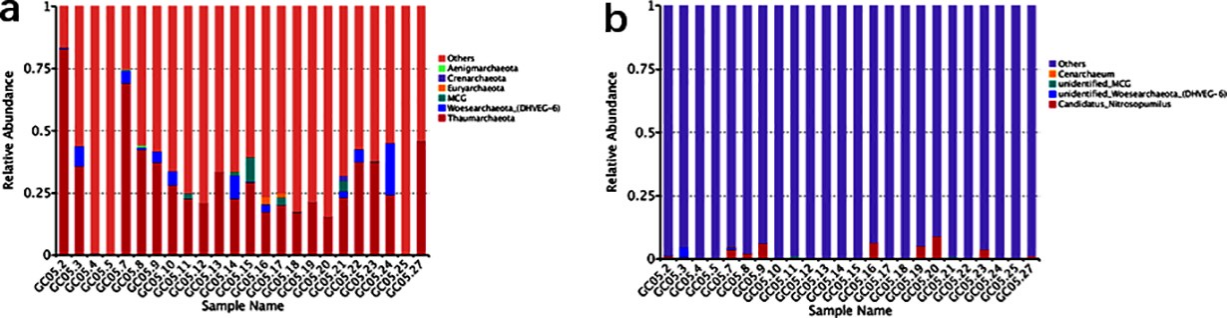
Relative abundance of dominant archaeal groups observed in sediments. Taxonomic distributions are depicted for the ranks of Phylum a) and Genera b). Each bar represents the relative abundance of each sample and each color represents a particular bacterial specie.

Moreover, for more detailed understanding of the predominant flora at different levels of taxonomic precision among each sample, we selected the maximum abundance of microbial taxa to make several histograms as follows (Figs 3 and 4).

From Figs 2A and 3, the majority of sequences were assigned to the phyla of Proteobacteria, Firmicutes, Actinobacteria, Bacteroidetes, Cyanobacteria and Chloroflexi. In other words, these 6 taxonomic groups were identified as the major bacterial taxa across all samples. Based on relative abundance, Proteobacteria was the most abundant bacterial phylum in these samples, especially in samples GC05.12, GC05.16 and GC05.22 with an average relative abundance of over 95% (Fig 3A). As the most dominant phylum, it had high affinities with four known classes: Gammaproteobacteria、Alphaproteobacteria、Betaproteobacteria and Deltaproteobacteria. Most of all, Gammaproteobacteria consisted widely and largely in all the samples, yet Alphaproteobacteria was distinctly rich at 20–80 cmbsf and 150 cmbsf (25.7% of bacterial reads on average) (Fig 3B). The second predominant phylum was Firmicutes, which accounted for 27.95% of the total sequences (Fig 2A). The majority of sequences belonging to Firmicutes were assigned to the two known classes—Bacilli and Clostridia. Bacilli also occupied a large proportion among these taxa assemblages, however, the relative abundance of the Clostridia only at the 130 cmbsf of this column was obviously higher than other depths (Fig 3B). In addition, the Actinobacteria were also widespread, but tend to be more abundant in the upper sediment layers (0–150 cmbsf), whereas the majority of Bacteroidetes was distributed in the deeper half of the column. Other phyla, such as Gemmatimonadetes, Saccharibacteria and Acidobacteria, were detected in most samples at relatively low abundances (Fig 3A).

In Fig 3C, at the Order level, Alteromonadales belonging to Gammaproteobacteria and Lactobacillales attaching to Bacilli were also spread in each sample with highly relative abundance, but it seems that Lactobacillales was richer after the 180 cmbsf sections. Whereas the relative abundance of Rhodobacterales which subordinating to Alphaproteobacteria in the upper 80 cmbsf of the column was much higher than in the remains latter layers. The pattern of distributions at this taxonomic level is highly congruent with the Class level mentioned above (Fig 3B). And among the 585 bacterial genera detected in this present study, the major genera included *Pseudoalteromonas*, *Lactobacillus*, *Profundibacterium*, *Escherichia-Shigella*, *Propionibacterium*, *Alteromonas*, *Enterobacter*, *Vibrio*, *Sphingomonas*, and *Staphylococcus* (Fig 3D).

For archaea data distributions exhibited visually in Fig 2B), a small fraction of the detected sequences belonging to 6 known archaea phyla while most of them still have been unannotated and unclassified by far. From the Fig 4A), the rarified sequences data set suggests that sediments archaeal communities from our site were overwhelmingly dominated by the phylum Thaumarchaeota regardless of GC05.4, GC05.5 and GC05.25. The phyla Woesearchaeota (formerly Euryarchaeota DHVEG-6 cluster) and Miscellaneous Crenarchaeotic Group (MCG, also known as Bathyarchaeota) [17] were widely distributed in several samples with slightly higher relative abundance. And the Euryarchaeota had more richness in GC05.16 and GC05.17 compared with other samples, but unfortunately, this phylum of sequences corresponding to anaerobic methanotrophic archaea were absent at our sampled station. At the level of genus (Fig 4B), *Candidatus Nitrosopumilus*, a known vital archaeal taxon, was investigated in most of the samples with an average 1.7% of total archaeal reads. And just like as we known, most archaeal sequences, especially in deep-sea sediments, have not been annotated complete taxonomic information up to now, hence that have failed to be classified into any phylum or genus from the above chart.

Other histograms of relative abundance of dominant species distributed at different taxonomic ranks were shown in S2 and S3 Figs.

### 3.4 Bacterial and archaeal community diversity

Diversity parameters of each sample, including community species richness (ACE, Chao1), diversity indices (Shannon, Simpson), and sequencing depth (Good’s coverage) were displayed in S2 and S3 Table. Good’s coverage estimator ranged from 98.3% to 99.9% for bacteria and 99.8% to 100% for archaea, indicating that high-throughput sequencing captured the majority of 16S rRNA gene sequences in each sample. ACE and Chao1 values revealed that bacterial richness was significantly higher than archaeal richness across all sediment samples. But there were less obvious trends among the Shannon and Simpson indices of diversity parameters in both bacteria and archaea assemblages.

**Fig 5:**
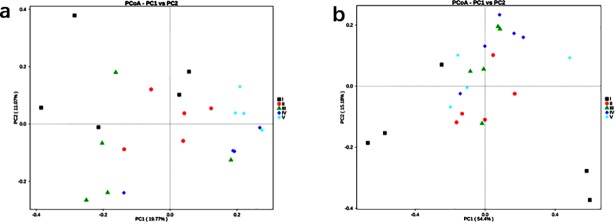
**Principal coordinate analysis (PCoA) plot on the weighted UniFrac distance matrix for bacteria a)/archaea b) community.** Different colors and shapes points represent different groups. The I group were GC05. 02 to GC05.07; The II group were GC05.8 to GC05.12; The III group were GC05.13 to GC05.17; The IV group were GC05.18 to GC05.22 and the V group were GC05.23 to GC05.27. Points that were closer together on the ordination have communities that are more similar.

For the sake of study if there are heterogeneities among various microorganisms at different depths intervals in the deep-sea REY-rich sediments, we divided all these samples into five groups depended on their original locations and then constructed the principal coordinate analysis (PCoA) using weighted UniFrac distance matrix for bacteria and archaea as shown in Fig 5.

No group was clustered together separately from Fig 5, which implicated that microflora distributed in our column did not vary greatly according to its vertical depth. Less evident taxonomic differences were observed along with the original depths of the samples, suggesting that microbial communities in our site may be less sensitive to the changes of depth. And considering the significant influences of environmental factors on the distributions of microbiota, such homogeneous sediments structures may be responsible for the vertical similarity of the microbial communities at the REY-rich sites, as revealed by PCoA analysis.

### 3.5 Bacterial and archaeal community associated with environment factors

**Fig 6:**
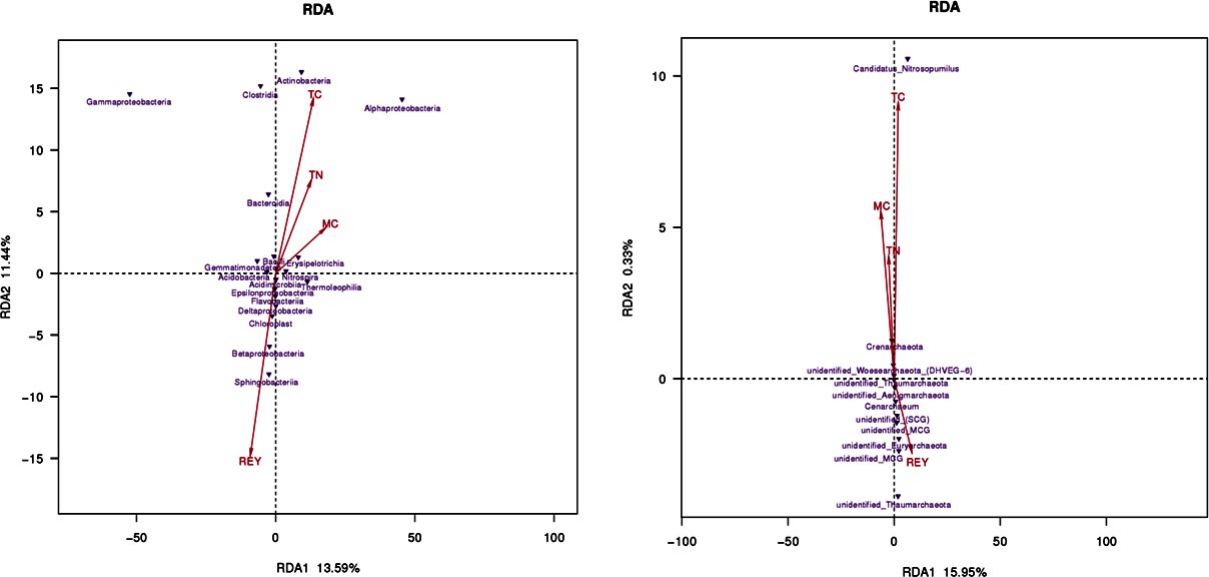
Redundancy analysis (RDA) of the relationship between the environmental factors and bacterial a)/archaeal b) flora composition. Arrows represent environmental factors.

As mentioned above, environmental factors were initially thought to be the dominant factors affecting variation among microbial communities. To discover the special microbial populations that may be influenced by the particular environmental variables, RDA analysis were carried out and the results showed a significantly negative correlation between nutritional factors (TN, TC, MC) with REY concentrations (Fig 6). Moreover, there were also other obvious trends, for examples, nutritional factors were strongly associated with some microbiota such as Gammaproteobacteria, Clostridia, Actinobacteria, Alphaproteobacteria and *Candidatus Nitrosopumilus*, while REY concentrations were positively associated with Sphingobacteria, Betaproteobacteria and many undefined archaeal taxa assemblages.

In relation to the linkage between microbial community diversity and sediments physical properties, Spearman correlation analysis was used to reveal the interactions (S4 Fig). The index of Shannon was positively correlated with MgO (Spearman r = 0.48, p<0.05) but negatively with Al_2_O_3_ (r = -0.46, p<0.05). And the estimators of species richness—observed species, ACE and Chao1 indices—were positively with Fe_2_O_3,_ MgO, MnO, V and Cu (all r>0.45, p<0.05) but were negatively with TC, SiO_2_ and Ba (all r<-0.46, p<0.05). Among the variables, MgO and Fe_2_O_3_ had the strongest influence on the diversities and abundances of the bacterial communities (S4 Fig). However, a dissimilar pattern appeared in the archaeal samples such that Spearman’s correlation test showed the Shannon and Simpson indices of the archaea were positively with TiO_2_ (r>0.57, p<0.01) while negatively with Cu (r<0.44, p<0.05) (S4 Fig).

## 4 Discussion

Microbial communities are vital components in deep-sea muds and play important parts in metal remineralization. Determining the composition and diversity of microbial communities in REY-rich muds of marine environments could provide valuable clues for abyssal rare earths mineralization researches. Here, we employed Illumina high-throughput sequencing of the 16S rRNA gene to survey the composition and diversity of REY-rich muds bacterial and archaeal communities in the central Indian Ocean basin and we suggest that various microorganisms may participate in the deep-sea REY mineralization.

In the current study, the most abundant bacteria were Proteobacteria, followed by Firmicutes and Actinobacteria. Within Proteobacteria, Gammaproteobacteria were dominant in all of samples and accounted for the largest proportion of bacterial sequences, which was consistent with previous studies of microbial communities in deep sea sediments [27–30]. Most researchers have found that the genera of *Escherichia* [31], *Pseudomonas* [32,33], *Shewanella* [33] and *Pantoea* [34] belonging to Gammaproteobacteria could adsorb REY ions onto cell wall from aqueous solutions, but the correlation between Gammaproteobacteria with REY concentrations in our results was not obvious. Compared with Gammaproteobacteria and Alphaproteobacteria, there were a relatively fewer sequencing assigned to Betaproteobacteria and Deltaproteobacteria. We found that the distributions of these two taxa were relevant to REY concentrations, which congruent with some previous studies. For examples, Takahashi et al. proved that *Alcaligene faecalis*, which belongs to Betaproteobacteria, also could adsorb REY ions [33]. And Merroun et al. reported that *Myxococcus*, a kind of bacterium of the Deltaproteobacteria, could accumulate 0.6 mmol of La/g of wet biomass and/or 0.99 mmol/g of dry biomass [35].

With respect to the second predominant phylum—Firmicutes, a majority of sequences were assigned to the class of Bacilli and Clostridia. Previous studies frequently reported that *Bacillus* belonging to the Class of Bacilli was capable of adsorbing more than 14 lanthanide REY [31,33,36,37]. However, in our study, the Bacilli were weakly associated with REY. Especially in the case of Clostridia, they seemed to be recalcitrant to the concentration of REY. In addition, the phylum Actinobacteria in our studied site seemed correlated strongly to the concentration of nutrient elements rather than REY, even if Tsuruta had testified that two genera, *Arthobacter* and *Streptomyces*, belonging to the phylum of Actinobacteria also could absorb REY [38].

Thaumarchaeota are one of the most abundant Archaea on the earth and has been detected in many kinds of environments such as soil, ocean and sediments [39–41]. Many microbes classified to the archaeal phylum Euryarchaeota were also detected in our study, in particular, the adsorption behavior of Eu (III) on *Halobacterium salinarum* was investigated in the past [42]. However, there were also so much archaeal sequences generated in our study not assigned into current known phyla or candidate groups, which emphasized that how little we know about archaea in deep sea muds up to now.

We also detected some other dominant taxonomic phyla in our samples. For examples, sequences belonging to the phylum Bacteroidetes comprised a partial proportion (3.92%) of the microbial community. Remarkably, our study results show that the Class of Sphingobacteria is strongly associated with REY, indicating that this microbiota may play important roles in the formation process of rare earth minerals. Additionally, we detected a large proportion of bacterial sequences belonging to the phyla Cyanobacteria. Similarly, Cyanobacteria have been observed in other dark deep-sea environments including South Adriatic Sea and North Pacific Ocean [43, 44]. Unfortunately, owing to insufficient experimental extents to date, it is impossible to determine whether these abundant phyla could have ability to absorb of REY. But we can infer that some bacteria, such as *Lactobacillus*, could reduce environmental pH and then create favorable environmental conditions for the enrichment of REY.

Up to now, little is known about the function of the vast majority of microbial groups in the central Indian Ocean REY-rich muds, thereby limiting our understanding of the microbial metabolic potential to the formation of rare earth minerals. Nevertheless, the current study provided the first baseline data of microbial communities in this area, which could serve as a foundation for further investigations into the relationship between microorganisms and rare earth minerals in this system. Further studies using RNA or other efficient approaches may reveal that microbial groups we identify in this study may drive nutrient cycling as well as influence the formation of rare earth minerals. And additional data is required to supplement and develop the theory that how microorganisms participate in the mineralization process of rare earths in the deep-sea sediments.

## Supporting information

S1 Table Sediments physicochemical properties of the studied site ^a^. (DOCX)

S2 Table High-throughput sequencing statistics and diversity measures for bacteria samples. (DOCX)

S3 Table High-throughput sequencing statistics and diversity measures for archaea samples. (DOC)

S1 Fig Rarefaction curves of bacteria(a)/ archaea (b) samples.
(TIF)

S2 Fig Relative abundance of dominant bacterial groups observed at rank of Family. Each bar represents the relative abundance of each sample. Each color represents a particular bacterial specie.
(TIF)

S3 Fig Relative abundance of dominant archaeal groups observed in sediments. Taxonomic distributions are depicted for the ranks of Class(a), Order(b) and Family(c). Each bar represents the relative abundance of each sample and each color represents a particular archaeal specie.
(TIF)

S4 Fig **The heat maps bacteria(a)/ archaea (b) indicating the relation between the environmental factors and alpha diversity index**. The environmental factors were longitudinal, the alpha diversity index was transverse. The value of Spearman correlation coefficient r is from -1 to 1, and r<0 was negatively correlated yet r>0 was positively correlated. The one or two asterisks in the heat map indicate that the correlation between diversity index and the specific factor is significant at the p < 0.05 and p < 0.01 levels, respectively.
(TIF)

S1 File Proof of approval.pdf. (PDF)
