# Supplementary material for: Microbial community composition and diversity in the Indian Ocean deep sea REY-rich muds
Source: PLoS One. 2018 Dec 17;13(12):e0208230. doi: 10.1371/journal.pone.0208230 (PMC6296507; doi:10.1371/journal.pone.0208230)
Supplement: S1 Table — (DOCX) [file pone.0208230.s001.docx]

**S1 Table. Sediments physicochemical properties of the studied site ^a^**

| **sample name** | **nutrients contents** | | |  | **major elements oxides contents** | | | | | | | | | |  | **partial trace elements contents** | | | | | | | |
| --- | --- | --- | --- | --- | --- | --- | --- | --- | --- | --- | --- | --- | --- | --- | --- | --- | --- | --- | --- | --- | --- | --- | --- |
|  | **TN** | **TC** | **TOC** | **MC**  **(%)** | **SiO_2_** | **Al_2_O_3_** | **CaO** | **Fe_2_O_3_** | **K_2_O** | **MgO** | **MnO** | **Na_2_O** | **P_2_O_5_** | **TiO** |  | **Ba** | **Sr** | **V** | **Cu** | **Ni** | **Pb** | **Co** | **Zn** |
|  | **(%)** | **(%)** | **(%)** |  | **(10^-2^)** | **(10^-2^)** | **(10^-2^)** | **(10^-2^)** | **(10^-2^)** | **(10^-2^)** | **(10^-2^)** | **(10^-2^)** | **(10^-2^)** | **(10^-2^)** |  | **(10^-6^)** | **(10^-6^)** | **(10^-6^)** | **(10^-6^)** | **(10^-6^)** | **(10^-6^)** | **(10^-6^)** | **(10^-6^)** |
| **GC05.1** | 0.13 | 0.22 | 0.16 | 0.75 | 45.8 | 13.25 | 1.54 | 9.05 | 2.67 | 3.2 | 2.49 | 6.62 | 0.61 | 0.63 |  | 1543 | 232 | 130 | 174 | 598 | 112 | 213.9 | 174 |
| **GC05.2** | 0.11 | 0.1 | 0.08 | 0.68 | 46.16 | 13.67 | 1.51 | 9.11 | 2.73 | 3.12 | 2.47 | 6.18 | 0.61 | 0.63 |  | 1357 | 223 | 126 | 396 | 552 | 109 | 214 | 168 |
| **GC05.3** | 0.12 | 0.09 | 0.09 | 0.72 | 44.68 | 13.98 | 1.62 | 9.96 | 2.64 | 3.29 | 2.74 | 5.84 | 0.72 | 0.69 |  | 1360 | 239 | 140 | 430 | 601 | 116 | 237.8 | 176 |
| **GC05.4** | 0.1 | 0.11 | 0.09 | 0.72 | 44.44 | 13.56 | 1.59 | 10.05 | 2.54 | 3.23 | 2.83 | 5.61 | 0.73 | 0.67 |  | 1491 | 237 | 145 | 443 | 651 | 129 | 255.1 | 171 |
| **GC05.5** | 0.1 | 0.13 | 0.09 | 0.71 | 44.42 | 13.8 | 1.6 | 10.17 | 2.59 | 3.31 | 2.85 | 5.55 | 0.73 | 0.7 |  | 1472 | 241 | 146 | 454 | 634 | 121 | 272.6 | 179 |
| **GC05.6** | 0.12 | 0.16 | 0.08 | 0.72 | 45.08 | 14.04 | 1.63 | 10.04 | 2.62 | 3.33 | 2.84 | 5.97 | 0.73 | 0.69 |  | 1433 | 244 | 147 | 432 | 652 | 116 | 264.9 | 181 |
| **GC05.7** | 0.11 | 0.18 | 0.1 | 0.74 | 45.6 | 12.93 | 1.47 | 8.85 | 2.63 | 3.03 | 2.43 | 6.14 | 0.6 | 0.6 |  | 1463 | 219 | 128 | 397 | 580 | 116 | 216.8 | 169 |
| **GC05.8** | 0.1 | 0.1 | 0.08 | 0.72 | 43.7 | 13.82 | 1.64 | 10.15 | 2.57 | 3.35 | 2.83 | 5.84 | 0.74 | 0.71 |  | 1420 | 241 | 144 | 439 | 648 | 118 | 267.9 | 179 |
| **GC05.9** | 0.1 | 0.11 | 0.07 | 0.71 | 43.7 | 13.81 | 1.63 | 10.04 | 2.6 | 3.22 | 2.85 | 5.48 | 0.74 | 0.68 |  | 1403 | 241 | 150 | 463 | 589 | 114 | 234.3 | 171 |
| **GC05.10** | 0.09 | 0.11 | 0.1 | 0.68 | 45.24 | 13.94 | 1.68 | 10.18 | 2.63 | 3.26 | 2.86 | 5.31 | 0.81 | 0.72 |  | 1396 | 243 | 146 | 417 | 628 | 119 | 267.3 | 176 |
| **GC05.11** | 0.1 | 0.1 | 0.07 | 0.67 | 44.58 | 14.13 | 1.68 | 10.15 | 2.64 | 3.22 | 2.84 | 5.2 | 0.82 | 0.72 |  | 1469 | 243 | 148 | 411 | 622 | 119 | 239.1 | 175 |
| **GC05.12** | 0.1 | 0.1 | 0.08 | 0.66 | 45.3 | 14.66 | 1.7 | 9.68 | 2.79 | 3.11 | 2.68 | 4.96 | 0.79 | 0.67 |  | 1438 | 235 | 149 | 426 | 588 | 109 | 227.5 | 168 |
| **GC05.13** | 0.09 | 0.12 | 0.1 | 0.65 | 44.96 | 14.07 | 1.81 | 9.82 | 2.76 | 3.11 | 2.78 | 4.72 | 0.9 | 0.7 |  | 1827 | 247 | 157 | 383 | 680 | 123 | 214.8 | 172 |
| **GC05.14** | 0.11 | 0.12 | 0.07 | 0.66 | 44.84 | 14.37 | 1.84 | 10.05 | 2.71 | 3.17 | 2.8 | 4.85 | 0.98 | 0.74 |  | 1481 | 243 | 145 | 397 | 667 | 117 | 268.4 | 184 |
| **GC05.15** | 0.08 | 0.07 | 0.06 | 0.66 | 44.36 | 14.37 | 2 | 9.99 | 2.75 | 3.15 | 2.86 | 4.9 | 1.1 | 0.73 |  | 1263 | 245 | 145 | 422 | 708 | 119 | 246.3 | 183 |
| **GC05.16** | 0.09 | 0.07 | 0.06 | 0.65 | 44.28 | 14.28 | 2.22 | 9.86 | 2.83 | 3.05 | 2.97 | 4.76 | 1.23 | 0.69 |  | 1107 | 249 | 161 | 425 | 776 | 130 | 242.2 | 184 |
| **GC05.17** | 0.07 | 0.07 | 0.05 | 0.65 | 43.2 | 14.16 | 2.57 | 10.05 | 2.95 | 3.09 | 3.17 | 5.09 | 1.52 | 0.66 |  | 830 | 259 | 151 | 522 | 860 | 118 | 299.9 | 198 |
| **GC05.18** | 0.06 | 0.05 | 0.05 | 0.65 | 42.62 | 14.19 | 2.9 | 10.38 | 3.04 | 3.12 | 3.41 | 5.23 | 1.78 | 0.64 |  | 680 | 271 | 158 | 593 | 925 | 120 | 317.8 | 208 |
| **GC05.19** | 0.08 | 0.06 | 0.05 | 0.66 | 42.36 | 14.1 | 3.12 | 10.54 | 3.01 | 3.18 | 3.61 | 5.35 | 1.9 | 0.64 |  | 713 | 283 | 160 | 577 | 1015 | 115 | 331.3 | 213 |
| **GC05.20** | 0.08 | 0.07 | 0.05 | 0.66 | 42.1 | 14.02 | 3.23 | 10.69 | 2.99 | 3.22 | 3.43 | 5.28 | 1.96 | 0.64 |  | 715 | 285 | 163 | 534 | 826 | 114 | 321.7 | 205 |
| **GC05.21** | 0.07 | 0.05 | 0.05 | 0.66 | 41.88 | 13.97 | 3.29 | 10.9 | 2.99 | 3.25 | 3.52 | 5.3 | 1.98 | 0.64 |  | 713 | 290 | 169 | 481 | 846 | 119 | 321.3 | 207 |
| **GC05.22** | 0.04 | 0.05 | 0.05 | 0.66 | 41.71 | 13.52 | 3.09 | 10.73 | 2.93 | 3.21 | 3.36 | 5.2 | 1.84 | 0.63 |  | 650 | 278 | 169 | 526 | 763 | 113 | 316.2 | 197 |
| **GC05.23** | 0.03 | 0.05 | 0.04 | 0.67 | 42.1 | 13.55 | 2.91 | 10.88 | 2.95 | 3.25 | 3.42 | 5.16 | 1.71 | 0.65 |  | 640 | 274 | 175 | 500 | 749 | 113 | 318 | 195 |
| **GC05.24** | 0.04 | 0.05 | 0.04 | 0.67 | 42.04 | 13.61 | 2.82 | 11.01 | 2.93 | 3.29 | 3.42 | 5.26 | 1.64 | 0.67 |  | 735 | 274 | 173 | 528 | 730 | 110 | 310.7 | 195 |
| **GC05.25** | 0.08 | 0.05 | 0.05 | 0.68 | 41.52 | 13.07 | 2.67 | 11.3 | 2.88 | 3.28 | 3.59 | 5.29 | 1.56 | 0.64 |  | 699 | 266 | 184 | 496 | 751 | 121 | 272.3 | 192 |
| **GC05.26** | 0.08 | 0.05 | 0.04 | 0.68 | 41.4 | 13.39 | 2.63 | 11.52 | 2.89 | 3.38 | 3.6 | 5.45 | 1.51 | 0.65 |  | 634 | 271 | 182 | 501 | 716 | 108 | 284 | 197 |
| **GC05.27** | 0.09 | 0.06 | 0.05 | 0.68 | 41.04 | 12.89 | 2.49 | 11.55 | 2.84 | 3.26 | 3.57 | 5.38 | 1.43 | 0.64 |  | 597 | 262 | 182 | 481 | 701 | 118 | 260.9 | 192 |

^a^TN, total nitrogen; TC, total carbon; TOC, total organic carbon; MC, moisture content.
