# Supplementary material for: Microbial community composition and diversity in the Indian Ocean deep sea REY-rich muds
Source: PLoS One. 2018 Dec 17;13(12):e0208230. doi: 10.1371/journal.pone.0208230 (PMC6296507; doi:10.1371/journal.pone.0208230)
Supplement: S2 Table — (DOCX) [file pone.0208230.s002.docx]

**S2 Table. High-throughput sequencing statistics and diversity measures for bacteria samples**

|  |  |  |  |  | **Alpha** | **diversity** | **Measures** |  |
| --- | --- | --- | --- | --- | --- | --- | --- | --- |
| **Sample**  **name** | **No. of sequences**  **reads** | **No. of observed OTUs** | **Observed species** | **ACE** | **Chao1** | **Shannon** | **Simpson** | **Goods'coverage** |
| **GC05.2** | 47,395 | 525 | 464 | 555.517 | 562.875 | 5.433 | 0.949 | 0.995 |
| **GC05.3** | 46,453 | 1,017 | 760 | 1,028.078 | 964.77 | 5.362 | 0.89 | 0.988 |
| **GC05.5** | 59,742 | 642 | 450 | 626.538 | 627.517 | 5.451 | 0.942 | 0.993 |
| **GC05.6** | 59,930 | 1,252 | 897 | 1,324.59 | 1,256 | 5.021 | 0.84 | 0.983 |
| **GC05.7** | 66,457 | 269 | 244 | 257.647 | 259.789 | 4.733 | 0.872 | 0.999 |
| **GC05.8** | 44,118 | 911 | 638 | 959.268 | 923.058 | 5.702 | 0.956 | 0.988 |
| **GC05.9** | 55,262 | 1,090 | 804 | 1,001.484 | 956.685 | 5.532 | 0.915 | 0.989 |
| **GC05.10** | 51,598 | 493 | 342 | 450.192 | 432.179 | 3.822 | 0.764 | 0.995 |
| **GC05.11** | 44,838 | 805 | 572 | 768.35 | 724.942 | 5.496 | 0.94 | 0.992 |
| **GC05.12** | 44,612 | 800 | 496 | 842.828 | 784.265 | 2.904 | 0.738 | 0.988 |
| **GC05.13** | 45,226 | 775 | 566 | 775.117 | 746.197 | 6.946 | 0.983 | 0.992 |
| **GC05.14** | 62,667 | 424 | 245 | 463.424 | 427 | 4.156 | 0.89 | 0.995 |
| **GC05.15** | 52,262 | 560 | 377 | 586.162 | 573.35 | 5.127 | 0.947 | 0.993 |
| **GC05.16** | 21,604 | 452 | 278 | 478.272 | 457.478 | 1.239 | 0.248 | 0.994 |
| **GC05.17** | 29,893 | 916 | 625 | 1,047.511 | 962.56 | 3.151 | 0.643 | 0.986 |
| **GC05.18** | 32,127 | 806 | 604 | 829.677 | 763.14 | 4.077 | 0.856 | 0.99 |
| **GC05.19** | 73,534 | 1,143 | 905 | 1,105.988 | 1,039.876 | 5.671 | 0.919 | 0.988 |
| **GC05.21** | 44,617 | 895 | 681 | 841.911 | 854.532 | 5.002 | 0.887 | 0.991 |
| **GC05.22** | 55,962 | 558 | 341 | 634.069 | 583.25 | 1.09 | 0.181 | 0.992 |
| **GC05.23** | 56,217 | 1,227 | 904 | 1,189.227 | 1,117.415 | 5.833 | 0.943 | 0.986 |
| **GC05.25** | 24,232 | 1,038 | 928 | 987.36 | 953.619 | 6.497 | 0.955 | 0.995 |
| **GC05.26** | 48,280 | 1,287 | 1108 | 1,262.445 | 1,215.551 | 6.782 | 0.958 | 0.989 |
| **GC05.27** | 50,826 | 1,100 | 948 | 1,119.196 | 1,057.091 | 6.124 | 0.945 | 0.989 |
|  |  |  |  |  |  |  |  |  |
| **Mean** | 48,602 | 825 | 616 | 831.95 | 793.18 | 4.833 | 0.833 | 0.991 |
|  |  |  |  |  |  |  |  |  |
| **Total** | 1,117,852 | 18,985 | 14,177 |  |  |  |  |  |
