# Supplementary material for: Microbial community composition and diversity in the Indian Ocean deep sea REY-rich muds
Source: PLoS One. 2018 Dec 17;13(12):e0208230. doi: 10.1371/journal.pone.0208230 (PMC6296507; doi:10.1371/journal.pone.0208230)
Supplement: S3 Table — (DOC) [file pone.0208230.s003.doc]

**S3 Table. High-throughput sequencing statistics and diversity measures for archaea samples**

|  |  |  |  |  | **Alpha** | **diversity** | **measures** |  |
| --- | --- | --- | --- | --- | --- | --- | --- | --- |
| **Sample name** | **No. of**  **Sequence**  **reads** | **No. of**  **observed**  **OTUs** | **Observed species** | **ACE** | **Chao1** | **Shannon** | **Simpson** | **Goods'coverage** |
| **GC05.2** | 45,575 | 263 | 233 | 280.575 | 297.474 | 3.953 | 0.869 | 0.999 |
| **GC05.3** | 61,132 | 337 | 291 | 340.98 | 340.111 | 5.414 | 0.944 | 0.998 |
| **GC05.4** | 56,793 | 170 | 170 | 264.037 | 296.067 | 4.332 | 0.92 | 0.998 |
| **GC05.5** | 57,159 | 162 | 137 | 165.392 | 151 | 4.778 | 0.949 | 0.999 |
| **GC05.7** | 50,986 | 200 | 185 | 208.148 | 210.375 | 4.482 | 0.914 | 0.999 |
| **GC05.8** | 52,043 | 188 | 166 | 198.799 | 187.12 | 5.022 | 0.951 | 0.999 |
| **GC05.9** | 66,451 | 200 | 163 | 207.934 | 200 | 4.872 | 0.95 | 0.999 |
| **GC05.10** | 59,322 | 162 | 135 | 165.738 | 159.167 | 4.941 | 0.956 | 0.999 |
| **GC05.11** | 53,778 | 226 | 200 | 240.8 | 249.4 | 5.653 | 0.969 | 0.999 |
| **GC05.12** | 69,372 | 162 | 134 | 162.123 | 153.118 | 5.055 | 0.956 | 0.999 |
| **GC05.13** | 57,040 | 178 | 153 | 173.282 | 165.158 | 5.229 | 0.96 | 0.999 |
| **GC05.14** | 37,600 | 225 | 179 | 221.561 | 197.026 | 5.021 | 0.956 | 0.999 |
| **GC05.15** | 43,812 | 214 | 178 | 219.724 | 209.889 | 4.882 | 0.943 | 0.999 |
| **GC05.16** | 62,292 | 211 | 175 | 222.197 | 218.333 | 5.394 | 0.958 | 0.999 |
| **GC05.17** | 61,835 | 167 | 146 | 175.521 | 162.111 | 4.655 | 0.942 | 0.999 |
| **GC05.18** | 35,403 | 164 | 137 | 179.085 | 162.5 | 4.439 | 0.912 | 0.999 |
| **GC05.19** | 57,216 | 219 | 185 | 251.058 | 256.87 | 4.116 | 0.861 | 0.998 |
| **GC05.20** | 34,464 | 242 | 191 | 242.482 | 224.781 | 4.743 | 0.933 | 0.999 |
| **GC05.21** | 49,484 | 219 | 178 | 234.13 | 234.895 | 5.064 | 0.953 | 0.999 |
| **GC05.22** | 55,986 | 178 | 162 | 200.936 | 197.286 | 4.687 | 0.938 | 0.999 |
| **GC05.23** | 51,141 | 245 | 208 | 249.276 | 241.176 | 4.501 | 0.923 | 0.999 |
| **GC05.24** | 68,586 | 181 | 146 | 203.265 | 180.44 | 4.472 | 0.93 | 0.999 |
| **GC05.25** | 36,862 | 200 | 167 | 224.221 | 210.385 | 3.915 | 0.896 | 0.999 |
| **GC05.27** | 58,971 | 173 | 163 | 176.143 | 180 | 5.201 | 0.951 | 1 |
|  |  |  |  |  |  |  |  |  |
| **Mean** | 53,471 | 204 | 174 | 216.975 | 211.862 | 4.784 | 0.935 | 0.999 |
|  |  |  |  |  |  |  |  |  |
| **Total** | 1,283,303 | 4,886 | 4,182 |  |  |  |  |  |
