# Supplementary figures and images for: Microbial community composition and diversity in the Indian Ocean deep sea REY-rich muds
Source: PLoS One. 2018 Dec 17;13(12):e0208230. doi: 10.1371/journal.pone.0208230 (PMC6296507; doi:10.1371/journal.pone.0208230)

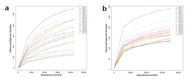

Supplement: S1 Fig — Rarefaction curves of bacteria(a)/ archaea (b) samples. (TIF) [file pone.0208230.s004.tif]

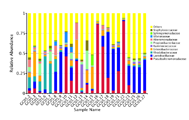

Supplement: S2 Fig — Each bar represents the relative abundance of each sample. Each color represents a particular bacterial specie. (TIF) [file pone.0208230.s005.tif]

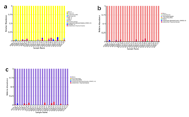

Supplement: S3 Fig — Taxonomic distributions are depicted for the ranks of Class(a), Order(b) and Family(c). Each bar represents the relative abundance of each sample and each color represents a particular archaeal specie. (TIF) [file pone.0208230.s006.tif]

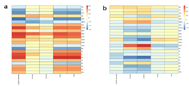

Supplement: S4 Fig — The heat maps bacteria(a)/ archaea (b) indicating the relation between the environmental factors and alpha diversity index. The environmental factors were longitudinal, the alpha diversity index was transverse. The value of Spearman correlation coefficient r is from -1 to 1, and r<0 was negatively correlated yet r>0 was positively correlated. The one or two asterisks in the heat map indicate that the correlation between diversity index and the specific factor is significant at the p < 0.05 and p < 0.01 levels, respectively. (TIF) [file pone.0208230.s007.tif]
