# Supplementary material for: Microbial community composition and diversity in the Indian Ocean deep sea REY-rich muds
Source: PLoS One. 2018 Dec 17;13(12):e0208230. doi: 10.1371/journal.pone.0208230 (PMC6296507; doi:10.1371/journal.pone.0208230)
Supplement: S1 File — (PDF) [file pone.0208230.s008.pdf]

中国大洋协会文件

大洋协发〔2014〕5号

中国大洋矿产资源研究开发协会关于执行中国大洋第34航次调查任务的通知

国家海洋局第二海洋研究所:

依据《国际海域资源调查与开发“十二五”规划》和《国际海域调查航次管理暂行办法》、中国大洋协会2014年工作计划，委托你所执行中国大洋第34航次调查任务，现将有关事项通知如下:

一、航次任务: 开展西南印度洋海域多金属硫化物合同区资源勘探工作,对重点区开展加密调查,同时兼顾生物与环境调查;在印度洋海盆开展稀土资源侦查性调查(以航次任务论证书方案一为准)。

二、航次执行时间: 2014年12月至2015年6月，航次任

务由“大洋一号”船执行。

三、航次组织实施：有关航次设计、航次实施方案和航次编队方案等事宜请按照《国际海域调查航次管理暂行办法》的有关规定执行；航次样品、资料和数据及现场施工管理请按照《大洋航次现场管理规定（试行）》、《大洋样品管理细则（试行）》等有关规定执行。

四、请你所尽快开展航次设计的编报工作。同时考虑到其它任务需求，做好有关任务调整的预案。

请你所统筹安排，加强组织，积极与船舶保障单位、相关参航单位联系，加大协调力度，科学、合理安排好航次人员、外事等工作，做好各项备航及航次组织实施工作。

中国大洋矿产资源研究开发协会

2014年6月5日

抄送：国家海洋局办公室（财务司）、国家海洋局国际司、国家海洋局第一海洋研究所、国家海洋局北海分局。

中国大洋矿产资源研究开发协会 2014年6月5日印发
